# Principal Semantic Components of Language and the Measurement of Meaning

**DOI:** 10.1371/journal.pone.0010921

**Published:** 2010-06-11

**Authors:** Alexei V. Samsonovic, Giorgio A. Ascoli

**Affiliations:** Structures, and Plasticity and Molecular Neuroscience Department, Center for Neural Informatics, Krasnow Institute for Advanced Study, George Mason University, Fairfax, Virginia, United States of America; University of California, Irvine, United States of America

## Abstract

Metric systems for semantics, or semantic cognitive maps, are allocations of words or other representations in a metric space based on their meaning. Existing methods for semantic mapping, such as Latent Semantic Analysis and Latent Dirichlet Allocation, are based on paradigms involving dissimilarity metrics. They typically do not take into account relations of antonymy and yield a large number of domain-specific semantic dimensions. Here, using a novel self-organization approach, we construct a low-dimensional, context-independent semantic map of natural language that represents simultaneously synonymy and antonymy. Emergent semantics of the map principal components are clearly identifiable: the first three correspond to the meanings of “good/bad” (valence), “calm/excited” (arousal), and “open/closed” (freedom), respectively. The semantic map is sufficiently robust to allow the automated extraction of synonyms and antonyms not originally in the dictionaries used to construct the map and to predict connotation from their coordinates. The map geometric characteristics include a limited number (∼4) of statistically significant dimensions, a bimodal distribution of the first component, increasing kurtosis of subsequent (unimodal) components, and a U-shaped maximum-spread planar projection. Both the semantic content and the main geometric features of the map are consistent between dictionaries (Microsoft Word and Princeton's WordNet), among Western languages (English, French, German, and Spanish), and with previously established psychometric measures. By defining the semantics of its dimensions, the constructed map provides a foundational metric system for the quantitative analysis of word meaning. Language can be viewed as a cumulative product of human experiences. Therefore, the extracted principal semantic dimensions may be useful to characterize the general semantic dimensions of the content of mental states. This is a fundamental step toward a universal metric system for semantics of human experiences, which is necessary for developing a rigorous science of the mind.

## Introduction

Words of natural language along with idioms and phrases are used in speech and writing to communicate conscious experiences, such as thoughts, feelings, and intentions. Each meaningful word, considered without any context, is characterized by a set of semantic connotations [1]. These connotations are a product of, and correlate with experiences communicated with the use of the word. Stated differently, communicated word semantics are behavioral correlates of experienced semantics. Therefore, the scientific characterization of word semantics can shed light on semantics of human experiences. In particular, if word meaning can be measured based on a metric system, the same metric system might be useful to measure the meaning of experiences. Thus, a precise metric system for the semantics of words could be a key in developing empirical science of the human mind [2].

To build a metric system for the semantics of words means to allocate words in a metric space based on their semantics, i.e., to create a semantic map of words. There are multiple ways to generate such maps based on the representation of semantic dissimilarity as geometrical distance [3]–[7]. Word semantics have multiple, possibly complementary aspects. Semantic maps created with distance metrics that emphasize different aspects may have different properties [8]. One aspect of word semantics determines the likelihood for the word to appear in a particular topic or document. Most of the previous studies devoted to allocating words in space based on their meaning, including Latent Semantic Analysis (LSA: [4]) and related techniques [5], focused on this aspect of word semantics, resulting in domain-specific semantic maps.

Here we develop an alternative approach based on the separate aspect of word semantics that determines whether two words are synonyms or antonyms (we will generally refer to a word that is either a synonym or an antonym as an *onym*). This aspect of word semantics, when expressed parsimoniously, is in many cases domain-independent, as may be illustrated with the following example. The term *short-term memory* belongs to the domains of cognitive, computational and neuro-sciences, together with its antonym: *long-term memory* [9]. At the same time, the general sense of the parsimoniously expressed antonymy relation, “short vs. long”, is applicable to virtually any domain.

This semantic aspect relates to the basic “flavor” of experience captured by generally applicable antonym pairs [10], [11]: e.g., *big* vs. *small*, *abstract* vs. *concrete*, *material* vs. *spiritual*, *whole* vs. *part*, *central* vs. *peripheral*, *one* vs. *many*, *rich* vs. *poor*, etc. Interestingly, we determined that the seemingly enormous variety of possible semantic directions is reducible to a small number (estimated as four) of main semantic dimensions that are in a definite sense orthogonal to each other. We found that these main semantic dimensions can be approximately characterized as (1) “good” vs. “bad”, (2) “calming” vs. “exciting”, (3) “open” vs. “closed”, and (4) “basic” vs. “elaborate”.

## Materials and Methods

### Linguistic Corpora and Core Dictionaries

This study was conducted using the dictionaries of synonyms and antonyms extracted from the thesaurus of Microsoft Office 2003 and 2007 Professional Enterprise Editions, further referred to as MS, in English, French, Spanish, and German, as well as the dictionary of English synonym and antonyms available as part of the Princeton WordNet 3.0 resource [12], further referred to as WN English or simply WN.

The MS corpora have independent origin for different languages (e.g., the English thesaurus was developed for Microsoft by Bloomsbury Publishing, Plc., while the French thesaurus is copyrighted by SYNAPSE Development, Toulouse, France). These MS dictionaries of synonyms and antonyms were acquired automatically with the following recursive procedure (see below for hardware and software details).

Step 1. Start in the thesaurus with the seed word “*first*”, or its translation in other languages. Alteration of the initial word never changed the resultant core dictionary by more than a few words.
Step 2. Add all synonyms and antonyms of the word to the dictionary, avoiding duplicates; repeat step 2 using each of these onyms sequentially as a new word.
Step 3. Take the next word from the thesaurus in alphabetical order, and repeat steps 2 and 3. After the last alphabetical word, resume with the first one and continue until the entire thesaurus is processed.

Next, we extracted the subset of the dictionary corresponding to the largest component of the graph of synonym and antonym links truncated to nodes (words) with a minimum of two links, including at least one antonym link, per node [13]. In particular, the MS dictionaries of synonyms and antonyms, and the equivalent WordNet dataset downloaded from the zipped files available online (http://wordnet.princeton.edu on 3/29/07), were further processed in the following ways.

Step 4. Symmetrize the onym relation by making all synonym and antonym links bi-directional. In other words, if word *A* is a synonym of word *B*, then *B* is synonym of *A*. This symmetrization is necessary to define the energy function.
Step 5. Eliminate onym inconsistencies: if word *A* is listed at the same time as synonym and antonym of word *B*, both onym relations between *A* and *B* are removed.
Step 6. Identify the largest connected cluster in the graph of onym relations. Remove all words that do not belong to this main cluster.
Step 7. Eliminate all words with no antonyms or fewer than two synonym/antonym links. The remaining dictionary of synonym and antonyms is referred to as the “core” dictionary.

The different core dictionaries had widely differing characteristics. The MS English core has 15,783 words, with an average of 11 synonyms and 2.7 antonyms per word. The WN English core has 20,477 words, with an average of 3.8 synonyms and 4.2 antonyms per word. The MS French core has 65,721 words, with an average of 6.5 synonyms and 10 antonyms per word. The MS German and Spanish cores have 93,887 and 259,436 words, respectively. The total size of each corpus is above 200,000 words, and in all cases, the extracted cores were a small part of the entire thesaurus. However, the next largest connected cluster was typically several orders of magnitude smaller than the core. For example, in WN the second largest connected cluster only contained 34 words.

### Construction of the Semantic Map

Our approach to constructing a cognitive map by self-organization of a distribution of words in a multidimensional vector space is inspired by statistical physics. At the beginning, we randomly allocate all *N* words of a given core dictionary as points in a high-dimensional unit ball, i.e. as vectors with length ≤1. The specific results described here were obtained with a dimension of 26, but they remained essentially identical when using the lower and higher dimension values of 10 and 100, respectively. Next, we minimize an “energy” or cost function *H* of the distribution, thereby finding a minimum or “ground state” of the system. The energy function of the word configuration **x**, was defined precisely as follows:
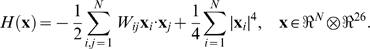
(*)


Here **x** *_i_* is the 26-dimensional vector representing the *i* ^th^ word (out of *N*) in the configuration **x**. The *W_ij_* entries of the symmetric relation matrix equal +1 for pairs of synonyms, −1 for pairs of antonyms, and zero for all non-onym pairs. Intuitively, maximizing the first sum moves synonyms towards the same hemispaces, while minimizing the second tends to align antonym pairs on opposite sides of the origin, reflecting their semantic relations. The fourth-power norm provides a soft limit to the absolute distance from the center. More specifically, the first term of the equation is the simplest analytical expression that captures the intent of aligning synonym vectors in parallel and antonym vectors in opposite directions. The last term is the lowest symmetric power term that is necessary to keep the distribution compact. This general approach and specific selection were empirically validated by their successful reconstruction of a map whose meaning was known a priori, that of color space, as illustrated at the end of the Results section.

This process may be illustrated with an example. In the initial random distribution of all words, before minimizing the energy function (*), the angles between word vectors in multi-dimensional space tend to be close to 90°. For instance, one specific simulation run using MS Word English data started from the following angles for a sample of word pairs: *right*/*wrong*, 63°; *excited*/*hectic*, 71°; *right*/*excited*, 91°. During the optimization process, words move from the initial random allocation based on their synonym/antonym relations, such that synonyms would “attract” each other and antonyms would “repel” each other. After the optimization is completed, the angles between the same word vectors become: *right*/*wrong*, 178° (almost opposite directions); *excited*/*hectic*, 12° (almost parallel); *right*/*excited*, 95° (almost orthogonal). These final angles do not depend on the initial angles.

The adopted optimization procedures included a second-order Newton algorithm using analytic expressions for derivatives of the energy function, and a zero-order steepest-descent algorithm with time-dependent “thermal noise” or simulated annealing. Convergence of the optimization was assessed by measuring the norm of the gradient of the energy function, as well as the relative change of the energy function itself and word coordinates in one iteration (see below for hardware and software details). In particular, the process was terminated whenever any of these monitored parameters fell below the threshold of 2·10^−6^ (dictated by the precision of calculations), which was achieved in all cases in less than 10^6^ steps.

When the optimization is completed, we rotate the resultant distribution to its principal components (PCs) by single value decomposition. Since the cost function and optimization procedure are symmetric with respect to the origin, the final sign of any PC coordinate is not meaningful by itself and can be considered a random outcome. Thus, upon completion of optimization, we flip each axis as needed to standardize its semantics for consistency among simulation runs. We selected the axes orientation arbitrarily once and for all maps, pointing the positive ends toward “good”, “exciting”, “open” and “elaborate”, respectively. Moreover, we normalized word coordinates by the average square length of all word vectors, effectively scaling the entire distribution to the unit variance. These post-processing operations of rotation, selective axis inversion, and rescaling, do not change the intrinsic shape of the optimized distribution, but are convenient and necessary for quantitative comparison of corpora.

The final distribution appeared to be systematically invariant with respect to the choice of initial random coordinates over multiple trials, suggesting that the global minimum of *H* (*) was reached in each case.

### Psychometric Data and Word Frequency Databases

The Affective Norms for English Words (ANEW [14]) database, developed by the Center for the Study of Emotion and Attention (CSEA) at the University of Florida, was kindly provided by Dr. Margaret M. Bradley. The ANEW database contains 1,034 words and was created using the Self-Assessment Manikin to acquire ratings of *pleasure*, *arousal*, and *dominance*. Each rating scale in ANEW runs from 1 to 9, with a rating of 1 indicating a low value (low pleasure, low arousal, low dominance) and 9 indicating a high value on each dimension.

Two word frequency databases were used. The first is the demographic (conversational) set from the British National Corpus (BNC), a 100 million word collection of language samples from a wide range of sources, representative of contemporary English. The XML Edition (2007 release), maintained by the University of Oxford (United Kingdom), was downloaded from http://www.natcorp.ox.ac.uk/corpus. The raw dataset distilled so to exclude those items occurring five or fewer times [15] included 14,736 words, of which 2,453 were common with the MS English core and were used in our study. The second word frequency database we employed is the Sydney Morning Herald Word Database, which contains frequency and density figures from one full year (1994) of newspaper publication, amounting to more than 23 million words in 38,526 articles. This “Australian” database, maintained by the University of Queensland, was downloaded from http://www2.psy.uq.edu.au/CogPsych/Noetica/OpenForumIssue4/SMH.html. The curator's filtering to exclude items that occur in only one article yield 97,031 words [16], of which 8,807 were common with the MS English core.

### Software and Hardware

The algorithm to acquire the MS dictionaries of synonym and antonyms (Steps 1–3 above) was based on COM (Component Object Model) automation, and implemented in MathWorks Matlab (v7.5, R2007b) following published examples [17]. The programs to extract the core dictionaries (Steps 4–7), to construct the semantic map (as described above), and to analyze the results, were custom implemented using a combination of GNU C (GCC 4.2) and Matlab along with their standard libraries and functions (all code is available upon request). These programs ran under the Windows XP Professional, Linux Fedora 7 and 8, or SunOS operating systems, either on a Dell Optiplex GX620 workstation or on a Sun Fire V890 server.

## Results

### Construction and Geometric Characterization of the Semantic Map

**Figure 1:**
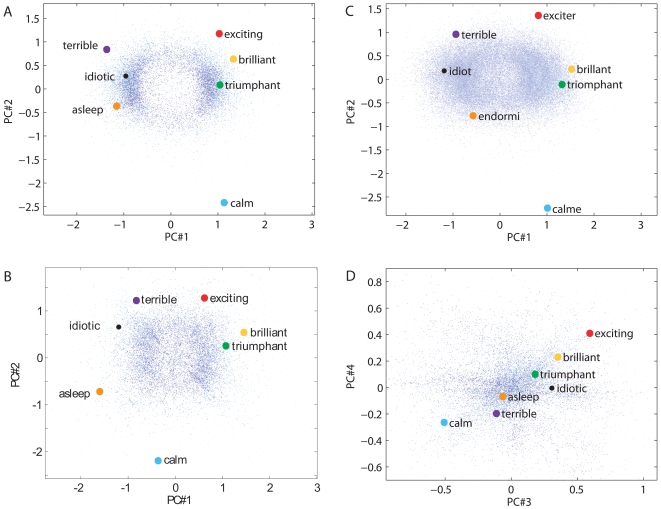
Principal components (PCs) of the constructed semantic map. Distributions of words in maximal-spread projections (PC2 vs. PC1) are shown in panels A–C. Coordinates are normalized by the squared-average vector length of all words. **A**: MS (Microsoft Word) English, **B**: WN (WordNet 3.0) English, **C**: MS French. **D**: MS English in PC3–PC4 coordinates. Representative words are labeled and identical terms or automated word-to-word translations are marked by same colors on different panels. The small blue dots represent all words of the corpora. A small random subset of words is plotted in light blue to aid visibility of individual dots in the face of excessive density (e.g., in panel C). Similarity of relative word positions is evident across panels A–C, but not D.

Starting from the synonym/antonym matrix extracted from the widely-employed English thesaurus of Microsoft Word (MS English), optimization converges to a definite stable state that is macroscopically independent of the initial random conditions (details in the Materials and Methods section above). Upon rotation to principal components and normalization to unit variance, the resulting spatial distribution of words displays distinct geometric features associated with corresponding word meanings, i.e. it constitutes a semantic map (Figure 1). A first semantic interpretation of the principal components was derived by examining the word sorted along each axis. The top and bottom of these lists indicated that the first principal component captures the notion of good/bad (‘valence’), the second of calming-exciting (‘arousal’), and the third of open-closed (‘freedom’). A more detailed semantic analysis is provided below.

**Figure 2:**
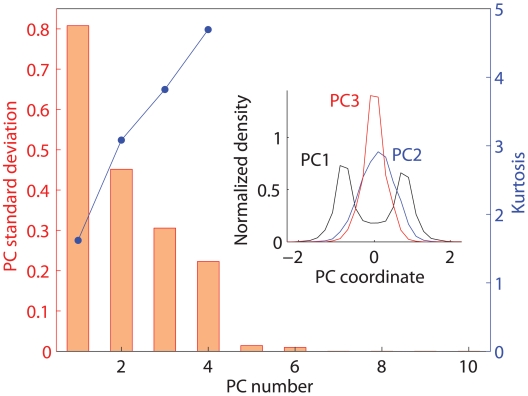
Standard deviations and kurtosis of the first PCs in the MS English map. **Inset**: distributions of word projections onto the first 3 PCs normalized to unit area under the curve.

The maximum spread planar projection (Figure 1A) exhibits a prominent “U-shape” resulting from a bimodal distribution along the first dimension and a unimodal distribution along the second. Subsequent components are all unimodal with a systematic increase in the “peakedness”, or kurtosis (Figure 2). The first three and four components encompass 95% and >99.9% of the spatial variance, respectively (Figure 2), irrespective of the dimensionality of the initial embedding (R^10^–R^100^).

Qualitatively similar features emerge when adopting an independent dictionary of synonyms and antonyms, Princeton's WordNet [12] (Figure 1B), and different languages, including French (Figure 1C), German, and Spanish (Materials and Methods section below).

### Qualitative and Quantitative Semantic Characterization of the Map

**Figure 3:**
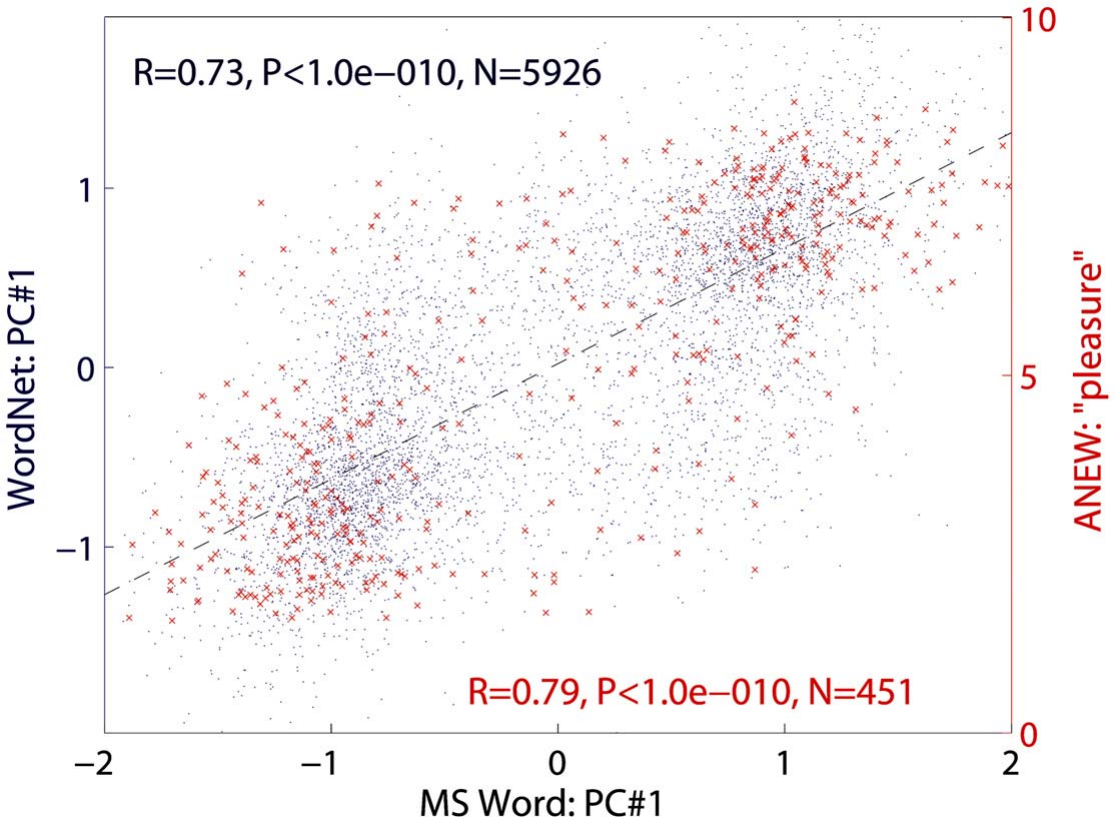
Semantic map correspondence across languages and methodologies. The scatter plots demonstrate numerical correspondence between MS English PC1 and both WN English PC1 (blue) and the first ANEW dimension, ‘pleasure’ (red). The dashed line represents the common linear fit. Captions show correlation coefficients (*R*), corresponding *P*-values, and numbers *N* of common words used for the analysis. All three distributions (MS English PC1, WN English PC1, and ANEW pleasure) are clearly bimodal. The correlations are highly significant even when analyzed for the two separate clusters of data. For words with negative MS English PC1 values, the correlation with the corresponding WN English PC1 values is R = 0.46 (p<10^−10^, N = 3101); and with ANEW: R = 0.36 (p<10^−7^, N = 226). For the positive MS English values, R = 0.40 for WN English (p<10^−10^, N = 2825) and R = 0.39 for ANEW (p<10^−8^, N = 225).

A key issue in the analysis of the constructed semantic map is the assignment of clearly recognizable semantics, if any, to each of the significant principal components, which are all geometrically orthogonal to each other. Such identification of the principal semantic components demonstrates the suitability of this approach to establish a metric to measure meaning and the content of mental states. The relative locations of words in the map consistently match the content of their meaning. Specifically, the projection of words onto the first principal component of the map systematically lines up along the “good-bad” dimension (‘valence’). More precisely, the sign of this coordinate robustly predicts the “positive-negative” content of each word, and the numerical value along this axis accurately orders words according to that aspect of their meaning. To illustrate this feature with an example, we ranked words describing mood (from best to worst) based on an independent psychometric measure of “pleasure” derived from a large number of human raters, namely the first of the Affective Norms for English Words (ANEW) [18]. Traversing the resulting list in the MS English map yields a quantitative “mood scale”, from *happy* (1.96), *confident* (1.50), *merry* (0.99), and *untroubled* (0.78), to *bored* (−0.57), *helpless* (−1.01), *hurt* (−1.33), *depressed* (−1.59), and *sad* (−1.89). These words follow the exact same order in the map derived from WordNet (WN), and the quantitative values between the two are tightly correlated (R = 0.95, p<10^−4^). This characterization of the first component generalizes to all words of the dictionary, demonstrating a highly significant correlation both between corpora (MS and WN) and with the ANEW ‘pleasure’ scale (Figure 3).

The second component of the map similarly orders terms based on a connotation of “calming-exciting” or “easy-difficult” (vertical axis in Figure 1A–C). Both the sign and the relative value of this coordinate are again consistent semantic predictors, as in the examples of *relax* (−1.55 in the MS map, −1.05 in WN), *troubling* (0.62, 0.95), and *excite* (0.99, 1.16). Since principal components are by construction orthogonal on the map, the values of word coordinates in these first two dimensions (PC#1 and PC#2) are mutually independent. In particular, words with negative ‘arousal’ value can be either good or bad, as in *soothing* (first principal component 0.69, second −1.19 in MS) and *boring* (−1.31, −0.94), and the same holds for positive arousal terms such as *thrilling* (0.88, 0.74) and *shocked* (−0.50, 0.76). More generally, while the positions of words in the maximum spread projection (first two components) are highly consistent among MS English, WordNet, and the map derived from MS French thesaurus (Figure 1A–C), they bear no implication on the values of subsequent components (Figure 1D).

The precise semantics of a component is given by the entire distribution of words on the map. For practical purposes, however, these semantics may be approximately described by the most representative words. In particular, the projection of a word on a given axis reflects its semantic amount along the corresponding component. At the same time, the alignment of a word with an axis provides an indication of the semantic specificity for that component. Thus, every semantic component of the map can be intuitively characterized by the words with both the largest projection on, and the best alignment with, each axis in either direction. For a given *i^th^* component, these words can be found as follows. We divide the *i^th^* coordinate of each word by the square root of their individual vector length, and sort all words according to the result. The projection of a word on each axis simply equals the value of the corresponding coordinate, while the alignment with an axis is measured by that coordinate value divided by the word vector length; thus the coordinate divided by the square root of the vector length is the geometric mean between the projection on, and the alignment with, a given axis. The top and bottom words of the sorted list are taken to represent the meaning of that component.

A similar process can be applied to antonym pairs. In particular, antonym pairs can be sorted by dividing the difference of the two words in the given coordinate by the square root of their vector distance. The top antonym pairs in the sorted list are also taken to represent the meaning of that component. Both approaches based on individual words and antonym pairs reveal definitive and consistent semantics for all four significant PC's in MS English (Table 1). For example, the top individual words for the first component (*clear*, *well…*, *improve*) all have positive valence, while the bottom ones (*decline*, *poor…*, *bad*) all have negative valence. Similarly, the sorted antonym pairs (e.g. *happy/sad*, *well/badly*, etc.) have opposite meaning relative to valence.

**Table 1:** Sorted lists of words and antonym pairs.

		PC #1	(valence)	PC #2	(arousal)	PC #3	(freedom)	PC #4	(mixed)
		*positive*	*negative*	*exciting*, *tough*	*calming, easy*	*close*, *dominate*	*open*, *free*	*rich*, *extra*	*basic*, *core*
**MS Word English**	**Individual words**	clear	decline	stiff	***calm***	**close**	***release***	later	basic
		**well**	**poor**	**hard**	relaxed	final	go	advanced	earlier
		accept	stop	**heavy**	mild	detain	fire	soggy	concise
		*praise*	uncertain	serious	**easy**	restraint	**free**	slowly	plain
		support	fail	*extreme*	gentle	**confine**	*freedom*	far ahead	quickly
		**good**	reject	deep	*modest*	swallow	independent	well ahead	crisp
		improve	**bad**	loud	**quiet**	restrain	**new**	far along	austere
	**Antonyms**	accept	decline	**hard**	soft	restrain	***release***	advanced	basic
		**good**	**poor**	fierce	***calm***	**close**	**open**	later	earlier
		*praise*	criticize	tough	**easy**	restraint	*freedom*	soggy	crisp
		well	**badly**	loud	**quiet**	restricted	**free**	wordy	concise
		*happy*	sad	**heavy**	insignificant	experienced	**new**	slowly	quickly
**WordNet**	**Ind. words**	**good**	ill	rough	smooth	close_up	**free**	inclined	disinclined
		bright	**bad**	stormy	***calm***	block	**open**	destroyed	unloving
		superior	**poor**	**heavy**	uncolored	bound	available	loving	outside
		animal	**badly**	**hard**	**easy**	covert	**new**	supportive	unsupportive
		**well**	unsaturated	wild	**quiet**	**confine**	unrestricted	encouraging	vertical
	**Antonyms**	**good**	inferior	rough	smooth	close_up	**free**	inclined	disinclined
		healthy	ill	dirty	***calm***	covert	**open**	loving	unloving
		intelligent	dull	**heavy**	fine	old	**new**	apt	vertical
		superior	**bad**	dark	***thin***	block	***release***	supportive	discouraging
		fit	unfit	troubled	**quiet**	**close**	leaky	encouraging	unsupportive
		tasty	**poor**	painful	**easy**	**confine**	phlegmy	synchronous	perpendicular
**Transl. French**	**I.W.**	*happy*	forgery	excessive	***calm***	catch	***to release***	neophyte	lost
		*praise*	weakened	*extreme*	*modest*	taken	*freedom*	fixed	monitor
	**Antonyms**	some	forgery	impetuous	***calm***	subjects	***release***	retained	gave up
		*happy*	cut down	enormous	*modest*	taken	to give up	neophyte	monitor
		to approve	to refuse	*extreme*	moderated	controls	delivered	subjugated	lost
		agreement	contradict	disproportionate	***thin***	tightened	smooth	bound	released

Each of the first 4 PCs for each of three corpora (MS English, WN English, MS French) is described by a list of the top and bottom individual words sorted by a combination of their projection on, and alignment with, the corresponding axis, as well as by similarly sorted pairs of antonyms. A total of 21 words are repeated within components, and they all involve the first 3 PCs: 13 between MS English and WN English (bold), 5 between MS English and MS French (italic), 1 between WN English and MS French and 2 among all three (bold italic). A total of 13 words are repeated across components, all except 1 involving PC4.

The semantics of the third and fourth orthogonal dimensions can be summarized as “open/closed” (‘dominance’) and “copious/essential”, respectively. The first three components, but not the fourth, are also consistent with the corresponding semantics of both the WN English corpus and the MS French corpus, after automatic translation into English with the Google translator tool (http://translate.google.com). In particular, a large number of terms repeated in the same components across corpora and languages, reflecting general semantic agreement in matching PCs (Table 1). As demonstrated in the next section, this correlation can be quantified and is statistically significant across these and several other languages and corpora. Words that ‘jumped’ components across corpora (*austere*, *bound*, *demolished*, *destroyed*, *dry*, *old*, *overcame*, *release*, *severe*, *slacken*, *subjected*, *subjugated*) always involve PC4, except one word (*smooth*) occurring in PC2 and PC3. Moreover, PC4 has no within-column cross-corpora repetitions, and in general shows lower consistency compared to the first 3 PCs.

**Figure 4:**
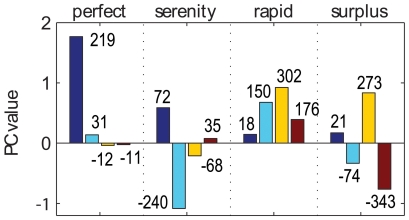
Values of the first four PCs for four different words in the MS English semantic map. PC coordinate values are represented in the bars, while the corresponding numbers express these quantities as percentages of the standard deviation of each PC (cf. Figure 2).

The general essence of each word can be thus quantitatively represented as a set of coordinates corresponding to its values along each of the principal components of the map (Figure 4). For example, the meaning of the word *serenity* has “good” valence (+0.59 on component 1), a major “calm” term (−1.08 on component 2), and a sense of “closure” (−0.21 on component 3). In this case, there is a clearly dominant component (the second). On average, by construction, the first components tend to have higher amplitudes than later components. This means that, broadly, the most informative element of a word is how “good” or “bad” it is, followed by how “calming/exciting”, etc. It is also interesting to compare the principal semantic components of a given word on a relative scale after filtering this general trend. This renormalization can be achieved by dividing each coordinate by the average amplitude of the corresponding component. In the *serenity* case, the third component becomes nearly as prominent as the first one on this relative scale (68% vs. 72%, respectively: Figure 4).

### Predictive Power of the Semantic Map

**Figure 5:**
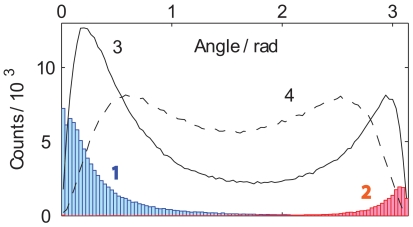
Angular distributions of word pairs on the map. The plots represent histograms of angle distributions for synonyms (1, blue), antonyms (2, red), onyms of onyms not listed as onyms (3, solid black line), and unrelated words (4, dashed line). Here “onym” stands for “synonym or antonym”, and onyms of onyms include synonyms of synonyms, synonyms of antonyms, antonyms of synonyms, and antonyms of antonyms.

As expected based on the form of the energy function *H*, words with similar meanings (synonyms) have similar proportions on the principal components of the map, i.e. small angles between their vectors (Figure 5). In contrast, words with opposite meanings (antonyms) tend to have anti-parallel vectors. In particular, synonyms and antonyms in MS English had median angles of 13° and 170°, respectively (means of 21° and 165°). Less than 3% of synonym pairs have angles greater than 90°, and less than 1% of antonyms have angles smaller than 90°. Upon checking, these exceptions revealed rare instances of questionable assignments in the source dictionary, which the map effectively “corrects”. For example, *opposite* and *harmonizing* are listed as synonyms in MS English, but their angle on the map, 145°, suggests otherwise. Although in most usage cases opposite and harmonizing would be considered antonyms (as predicted by the map) the assignment as synonym in the source dictionary may still be appropriate in specific contexts (such as in describing power balance, or musical tones). As an alternative example, hot and cool are typically antonyms (referring e.g. to weather or beverages), except when used idiomatically to describe an idea, a videogame, or a classmate.

Overall, given a pair of synonyms or antonyms in the dictionary, their dot product identifies the correct “onyms” relation with 99% accuracy. In particular, four real numbers associated with each word contain all essential information to identify antonyms among related terms: all semantic flavors of antonymy are reducible to four principal semantic dimensions. In contrast, random pairs (i.e., typically unrelated words) have an average angle of 90°, with less than 3% of values below 13° or above 170°.

It is tempting to extrapolate these considerations and assume that proximity of two words in the map is sufficient to ensure a similarity of their meanings. However, this is not the case. Unrelated word pairs vastly outnumber synonyms (∼1500∶1) and antonyms (∼7400∶1). The majority of unrelated words pertains to separate semantic domains, and could not possibly be considered synonyms or antonyms. Even the tail ends of their angle distribution constitute a disruptive confounder of the semantic relations. Stated differently, given a particular word, it is fair to assume that, among all *related* terms, synonyms will be concentrated in the neighborhood and antonyms in the antipodes. Nevertheless, unrelated words will still constitute the majority of terms even close to 0° and 180°. These unrelated words randomly end up in the proximity of a given term by virtue of their large number in the self-organizing reduction of the high number of initial dimensions into the low-dimensional principal component space. Therefore, the constructed semantic map of words differs from the high-dimensional semantic spaces typically obtained with other existing approaches [4], [19], in which the distance between any pair of embedded symbols reflects the whole semantic dissimilarity for a restricted contextual domain. Our low-dimensional map complements those local approaches by observing global semantic properties. Here, the distance between locations selectively measures the aspects of the dissimilarity broadly applicable to any context, without distinguishing between domain-specific semantic flavors.

A pool of terms likely related to a given word is constituted by all synonyms of synonyms or, more generally, “onyms of onyms” of that word. In particular, words which are onyms of onyms are usually in overlapping semantic domains, but not all words in overlapping domains are onyms of onyms. Having a synonym or antonym in common does not guarantee, but strongly indicates, that two words pertain to overlapping semantic domains. Thus, within the pool of onyms of onyms, one could expect angular information to be a powerful predictor of semantic content. To test this hypothesis, we sampled 20 words from MS English and WN English, and computed the cosines of their angle with each of their onyms of onyms. We then assigned the binary values of +1 and −1 to the onyms of onyms that were also reported as synonyms or antonyms, respectively. The correlation between the cosines and binary values was statistically significant in all 40 cases (Table 2).

**Table 2:** Assignment of synonyms/antonyms among related words.

*Corpus*	*MS English*			*WN English*		
Word	N	*R*	*P*	N	*R*	*P*
above	232	0.92	1.8·10^−8^	67	1.00	4.1·10^−22^
below	122	1.00	2.4·10^−25^	64	1.00	2.2·10^−27^
good	2342	0.98	7.0·10^−70^	3470	0.97	7.8·10^−140^
bad	1760	0.85	2.3·10^−35^	2903	0.96	1.0·10^−85^
exciting	665	0.99	1.9·10^−45^	199	1.00	1.9·10^−16^
calming	296	0.89	7.3·10^−7^	74	0.93	4.9·10^−13^
open	2271	0.95	1.5·10^−83^	2673	0.95	1.0·10^−82^
close	3077	0.78	1.3·10^−34^	2759	0.96	1.2·10^−88^
voluntary	328	0.83	4.8·10^−7^	229	0.89	5.0·10^−17^
basic	1105	0.76	4.4·10^−12^	489	0.78	2.5·10^−12^
central	502	0.98	5.8·10^−29^	1217	0.91	2.4·10^−10^
peripheral	215	0.92	2.5·10^−6^	350	0.88	1.4·10^−11^
take	3219	0.40	4.3·10^−7^	1096	0.68	7.4·10^−28^
give	2197	0.39	3.2·10^−4^	1005	1.00	3.1·10^−7^
increase	2111	1.00	8.2·10^−154^	228	1.00	1.2·10^−20^
decrease	1317	0.98	4.4·10^−56^	88	1.00	2.1·10^−19^
boring	834	0.97	8.6·10^−36^	310	1.00	1.8·10^−21^
soothing	445	0.95	2.4·10^−15^	68	1.00	1.4·10^−9^
catastrophic	128	1.00	<10^−256^	96	1.00	8.2·10^−8^
triumphant	207	0.98	2.5·10^−12^	70	1.00	6.0·10^−8^

Assignment of synonyms/antonyms among related words. N is the number of onyms of onyms of each listed word in either corpus. *R* is the correlation coefficient between the dot product of the of onyms of onyms with the original listed word, and a binary value indicating if each onym of onym is listed as a synonym (1) or antonym (−1) of that word. *P* is the probability to obtain such correlation by chance.

In addition to finding systematically significant numerical values in all 40 cases examined, this compilation reveals the consistent ability of the map to identify, based on the dot products, “new” synonyms and antonyms not explicitly listed as such in the dictionary. A specific example may constitute a useful illustration. In WN, the term *antonym* has 22 onyms of onyms. Among these, the two terms with the largest positive dot products are the only listed synonyms, namely *opposite_word* (1.000) and *opposite*. Similarly, the words with the largest negative dot products are the only two listed antonyms, namely *equivalent word* (−0.999) and *synonym* (−0.998). The two onyms of onyms with the positive and negative dot products closest to zero lack any synonym/antonym content: *cyclic* (0.190) and *secondary* (−0.066). These qualitative observations are reflected in an *R* value of 1.00 and a *P* value of 3.3·10^−8^.

The term *antonym* is not part of the MS English core, but the word *opposite* is, and has 306 onyms of onyms. In this case, however, the same analysis returns relatively weaker *R* and *P* values of 0.44 and 0.025, respectively. A closer inspection to the list of onyms of onyms explains this apparent inconsistency and further corroborates the predictive value of the semantic map. The top ranking positive dot products correspond to terms listed as synonyms, namely *dissimilarity* (1.00), *the other extreme*, and *contra* (both 0.98). Next in the list, while not reported as synonyms, are nonetheless correct predictions: *heretical*, *heterodox*, *competing*, and *contrary to accepted belief* (all 0.98), followed by *contending* and *hotheaded* (both 0.97). Interestingly, the next terms at similar values are again listed as synonyms: *inverse*, *opposing* (both 0.97), *deviating*, and *contrary* (both 0.96).

The lower correlation value for *opposite* in MS is due to a few outliers, such as *harmonizing* (dot product of −0.80, but listed as synonym). As discussed above (see footnote 1), even in these cases the map intuitively appears to be robust enough to actually “correct” mistaken assignments (i.e., *harmonizing* is more akin to an antonym than a synonym of *opposite*). To quantify this impression, we computed the correlation for the subset of the onyms of onyms that are listed as synonyms or antonyms of the word *opposite* in the independent WN dictionary, but not in MS. In other words, we “tested” the predicted assignment of the MS semantic map based on the available data in the WN dictionary. The resulting *R* and *P* values (0.99 and 0.005) were statistically significant, and the identified terms were consistent both among the new synonym (*different*, dot product of 0.94) and new antonyms (*like*, *similar*, and *same*, at −0.74, −0.93, and −0.94, respectively). Furthermore, the words with even more extreme negative dot products, although not explicitly listed in either dictionary, were all consistent with antonym meanings: *resemblance*, *congruence* (both −0.96), *analogy* (−0.97), *equivalence*, and *similarity* (both −0.99).

**Figure 6:**
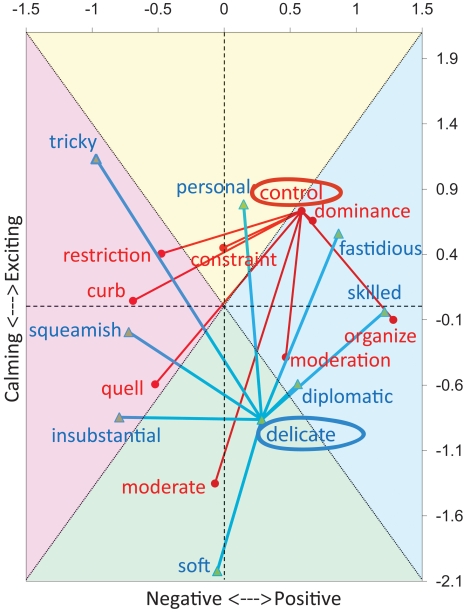
Semantics of the cognitive map (MS English): examples of connotation mapping. For each of the two representative (bold and circled) words, *control* and *delicate*, 8 synonyms are selected such that they nearly uniformly occupy all quadrants.

A potential practical application of the described semantic map consists of specifying the connotation as well as the general meaning (denotation) of words. An illustration of considering connotation is provided in Figure 6, where onyms of onyms of two words (*control* and *delicate*) are plotted in the plane of the first two principal components. In general, terms are located in the proper octant according to the connotation of their meaning (“good”, “good/exciting”, “exciting”, “bad/exciting”, “bad”, “bad/calming”, “calming”, “good/calming”). For instance, the term *control* can be substituted with a “good” connotation by *organize*, or with a “bad” connotation as *curb*. Likewise, *delicate* can connote a “calming” semantic as *soft* or an “exciting” semantic as *personal*.

Moreover, the vector representation of words in this map has both absolute and relative meanings. For example, the terms *okay* and *good* lie in the same quadrant of the map with an angle of 10° between them and can be considered “absolute” synonyms. In particular, they both have a positive value in the first component (1.36 and 2.13, respectively). However, with respect to the position of *fine*, these two terms lie on opposite sides (i.e., the angle between the vector connecting *fine* and *okay* and that connecting *fine* and *good* is greater than 90°). Relative to *fine* (whose value in the first component is 1.70), the term okay has actually a negative valence (−0.34), whereas the term good has a positive one (0.43).

**Figure 7:**
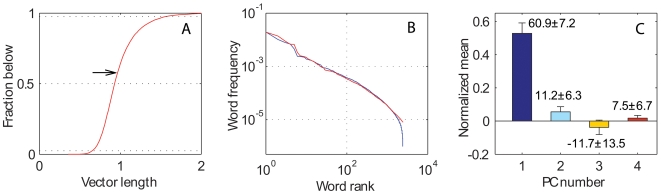
Semantic characteristics of the frequency of word usage. **A**: cumulative distribution of vector length of all words in MS English, with dotted horizontal lines at the 2.5^th^, 50^th^, and 97.5^th^ percentiles. The arrow indicates the mean weighted by the British National Corpus (BNC) frequency distribution. **B**: MS English word sorting by the frequency of their usage according to two independent sources (see Materials and Methods): Australian database (blue) and BNC (red). **C**: Values of the first 4 PCs of the weighted average of all words according to the Australian database frequencies. As in Figure 4, the bars and corresponding numbers represent the PC coordinate values and their percentage of the standard deviation of each PC (in the case of BNC frequencies, the corresponding numbers are: 64.0+7.5%, 13.3+6.4%, −15.4+11.9%, and 10.2+6.4%). Standard errors are reported for both bars (as whiskers) and numbers. Only the first component is statistically significant.

The length of the vector can also be interpreted as a measure of the semantic component of a word measured by its main map dimensions, i.e. the aspect of the word meaning that distinguishes between antonym and synonym relations across most contexts. For example, the term *relevant* has greater vector length (1.33) than the term *pertinent* (1.15), but smaller than the term *important* (1.90). The word closest to the center is *emigrant* (vector length 0.36). Despite its definite meaning, this word is relatively neutral with respect to the main semantic dimensions of the map. The distribution of lengths over the whole dictionary (Figure 7A) shows a median meaning of 0.93 (μ±σ = 0.98±0.23). In contrast, the average semantics of the dictionary computed as the vector mean of all words nearly coincides with the origin of coordinates, i.e. the point of “no meaning” (first three components: −0.033±0.006, 0.080±0.004, and 0.004±0.002).

However, words have different usage frequency in language (Figure 7B). For example, the term *doctor* (which is used on average every 5511 words) is 26 times more common than the term *professor*. It is thus possible to compute an overall “concept mean”, as the frequency-weighted average position of all words in the semantic map. Such measure captures the most representative meaning composed across a particular language (Figure 7C). In English, this vector has a significant length (close to 0.5) and a non-uniform contribution of principal components. In particular, the significantly positive projection on the first axis (>0.5) corresponds to a “good” semantic, while all other dimensions have non-significant values. The same holds for the difference between the frequency-weighted and the absolute vector means. Thus, positive words are used more frequently in English than negative words (*P*<10^−18^), while there is no significant preference in the other semantic dimensions (all three *P*>0.3).

### Statistical Cross-Corpus Semantic Comparison

The semantic characterization of the map principal components also enables a direct comparison across corpora, languages, and data types. As mentioned earlier, the first three components, but not the fourth, demonstrate high consistency across independent corpora (MS English vs. WN English) and languages (cf. MS French, Table 1). To extend the comparison of principal semantic components to a quantitative measurement across additional corpora and languages, we also Google-translated the MS German and MS Spanish dictionaries into English. For the scope of this analysis, each corpus (after translation as applicable) was limited to the set of words that overlapped with the MS English core dictionary. For example, the 15,783 MS English core words and the 20,477 WN English core words have 5926 terms in common. For MS French, the overlap was 4704 English words, mapped onto from 19,944 French terms, representing approximately 30% of the MS French core dictionary. Many French words projected onto single English words, because word inflections are listed separately in the MS French thesaurus; the same occurred in German. We then extracted several correlation measures between the word coordinates from each of the separate semantic maps (WN English, MS French, MS German, and MS Spanish) and the MS English map.

First, for each pair of corpora, we computed a matrix of PC-to-PC correlation coefficients (Table 3). Results demonstrate a systematic two-way semantic correspondence of the first three PCs for all compared pairs of corpora. In particular, each of the first three PC in every corpus displays the highest correlation coefficient with the corresponding PC of the other corpus in the pair. These values are all statistically significant (p<0.001). Such correspondence only holds for the fourth component between MS English and WN English, but not across different languages. Dimensions beyond the fourth are not statistically significant in MS English and are thus not represented in this table. Moreover, we compared the first three PCs of MS English with the three original dimensions of ANEW, whose semantics are identified as *pleasure*, *arousal*, and *dominance*. In this case, the two-way semantic correspondence was only revealed on the first two components. This is not surprising given that the coordinates of the ANEW dataset are not internally orthogonal. In fact, the first and third coordinates are highly correlated within the ANEW sample. We also computed the correlation of the first 4 MS English PCs with each of the 32 Paivio norms [20] and of the 51 Rubin properties [21], which constitute, to the best of our knowledge, the largest available collections of psychometric measures. However, none of these attempts resulted in higher correlation coefficients than those found for ANEW.

**Table 3:** Correlations of word coordinates across corpora.

			MS English					
		PC1	PC2	PC3	PC4	CCA	Other parameters	
WN English	PC1	**0.73**	0.20	−0.06	−0.031	0.78	5926	a
	PC2	−0.23	**0.64**	0.18	0.22	0.72	7	b
	PC3	0.12	−0.13	**0.57**	0.13	0.63	6.6·10^−4^	c
	PC4	0.029	−0.022	0.001	**0.30**	0.52	0.76	d
MS French	PC1	**0.74**	0.0057	0.0004	0.034	0.75	4704/19944	a
	PC2	−0.01	**0.41**	0.24	0.14	0.54	7	b
	PC3	−0.034	−0.33	**0.37**	0.0097	0.49	2.0·10^−2^	c
	PC4	0.056	0.066	−0.0058	0.021	0.27	0.74	d
MS German	PC1	**0.73**	0.037	0.025	0.056	0.78	5290/35464	a
	PC2	−0.081	**0.21**	0.16	0.097	0.57	6	b
	PC3	0.049	−0.16	**0.26**	0.029	0.46	1.1·10^−4^	c
	PC4	−0.089	0.007	0.014	0.026	0.24	0.73	d
MS Spanish	PC1	**0.67**	0.037	−0.046	−0.014	0.71	1269/1269	a
	PC2	−0.20	**0.45**	−0.13	0.14	0.62	7	b
	PC3	0.17	−0.056	**0.46**	0.066	0.60	0.005	c
	PC4	0.0014	0.33	0.19	0.18	0.45	0.68	d
ANEW	D1	**0.80**	−0.19	0.20	0.21	0.83	451	a
	D2	0.052	**0.39**	0.26	0.22	0.55	2	b
	D3	0.0085	0.22	0.094	−0.22	0.37	<10^−10^	c
							0.80	d

Correlations of word coordinates across corpora. MS English dictionary is correlated with WN English, translated MS French, MS German, and MS Spanish, as well as the ANEW database. PC1–PC4 represent the first 4 principal components (D1–D3 are the 3 non-orthogonal dimensions of ANEW), and the numbers in each column are the corresponding correlation coefficients. The correlation coefficients with the consistently highest absolute values within their row and column (if any) are typeset in bold. CCA: the first four canonical correlation coefficients. Other parameters (right column), **a:** the number of common words in each pair of corpora (English/foreign); **b:** the number of significant canonical correlation components; **c:** the *P* value of the last significant component (all *P* values of the previous components are smaller); **d:** the overall correlation (**) of the compared corpus pair. All values reported in the Table are statistically significant.

Next, we subjected each pair of corpora to canonical correlation analysis [22] (CCA). CCA finds the basis vectors for two sets of multidimensional variables such that the correlations between the projections of the variables onto these basis vectors are mutually maximized. The first four CCA coefficients are reported in Table 3 for each pair of corpora. CCA rotates two distributions of points so as to align them for maximal correlation. Thus, the first CCA correlation must be, by construction, higher than (or equal to) the correlation between the first principal components independently obtained in the two sets. The fact that these values are extremely close between MS English and each of the other corpora (e.g. 0.78 vs. 0.73 for WN English, 0.75 vs. 0.74 for MS French, 0.83 vs. 0.80 for ANEW) suggests an excellent alignment of their intrinsic principal components. Moreover, the fact that the number of statistically significant canonical correlations (7 for WN English, French, and Spanish, and 6 for German) systematically exceed the number of significant dimensions in MS English (4) is a further indication of geometric consistency across corpora, even if the semantics no longer strictly correspond beyond the fourth dimension.

Finally, as an additional method of quantifying the linear relationships between pairs of corpora (i.e., two multidimensional variables), we defined an “overall correlation” *OC* (**) based on the norms of the covariant matrices, which are the natural generalization to higher dimensions of the concept of the variance of a scalar-valued random variable. The covariance matrix or dispersion matrix is a matrix of covariances between elements of a vector, and naturally generalizes to higher dimensions the concept of the variance of a scalar variable. The correlation coefficient for a pair of scalar variables is the ratio of their covariance to the product of their standard deviations. Our formulation (**) is a natural extension to variables in multiple dimensions. The formula is analogous to that of the Pearson correlation coefficient, and coincides with it in one dimension:
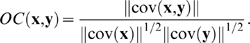
(**)


This measure characterizes the alignment of two distributions of points, each independently rotated to their internal principal components, throughout all of their dimensions. The overall correlation coefficient consistently assumed high values (between 0.68 and 0.80), always intermediate between the first canonical correlation and the correlation between first principal components.

This result of the cross-corpus comparison, as well as the qualitative assessment of the semantic content of the significant principal components, also proved to be generally robust with respect to alterations of the cost function parameters and/or the initial conditions in optimization. These findings indicate overall consistency and reliability across languages, datasets, and variations of the technique.

### Validation in Color Space

To verify the general applicability and robustness of our approach, we designed a simple simulation of color mapping. The model semantic space *X_color_* was defined as a sphere *S^2^*, in which each point was associated with a unique color, using the three Cartesian coordinates as RGB values. A number *n* of points (initially set to *n* = 1000) were randomly sampled from *X_color_*. For each sampled point, a list of “synonyms” and “antonyms” was generated by stochastically selecting neighbors within a certain ‘threshold angle’ as synonyms and neighbors within that threshold angle from the antipode as antonyms. The initial values for the threshold angles and the average number of onyms per point (the ‘degree’ of the graph) were set to 20° and 3.5, respectively, consistently with the parameters of the available linguistic corpora, and later allowed to vary as described below.

**Figure 8:**
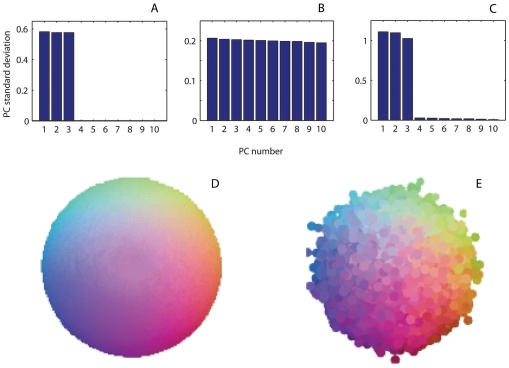
Reconstruction of the color map. **A**: original PC standard deviations in *d* = 10. **B**: standard deviations of PCs in the starting configuration selected for optimization. **C**: reconstructed PC standard deviations in *d* = 10. **D**: original color space map. **E**: reconstructed color space map.

The points were then embedded in a *d*-dimensional space (with a default value of *d* = 10) with random initial coordinates. Their coordinates were optimized by minimizing the above-described energy function *H* of locations and synonym-antonym connections, using the same convergence criteria adopted for the main language study (see ‘Construction of the Semantic Map’ in Materials and Methods). Finally, the resultant distribution was rotated to principal components (Figure 8). The resulting accurate reconstruction of the coloring of the sphere indicates that the topology and geometry of this cognitive map (whose semantic was in this case known by construction) could be reconstructed from a sparse subset of synonym and antonym relations.

**Figure 9:**
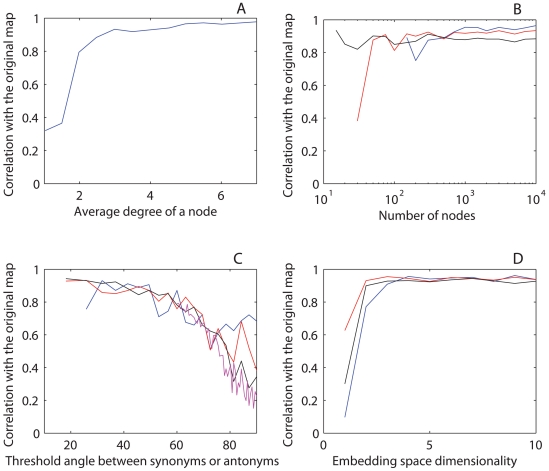
Robustness of the color map reconstruction. **A**: correlation between the reconstructed map and the original map as it varies with the embedding space dimension *d* for three different values of the threshold angle between “onyms”: 10° (blue), 20° (red), and 30° (black). The number of nodes and their average degree are 1000 and 3.5, respectively. **B**: correlation between the reconstructed and the original map as a function of the average node degree. The number of nodes, embedding dimension, and threshold value are 1000, 10, and 0.90, respectively. **C**: correlation with the original map as a function of the number of nodes. The embedding dimension, threshold, and average degree are 10, 0.50, and 3.5, respectively. **D**: correlation with the original map as a function of the threshold angle between “synonyms” and “antonyms” for four different values of the number of nodes: 100 (blue), 300 (red), 1000 (black), 5000 (magenta). The embedding dimension and average degree are 10 and 3.50, respectively.

Specifically, after reconstruction, the amplitudes (standard deviations) of the first three PCs are each close to 1, while the remaining 7 are negligible (Figure 8C), resembling the situation observed in MS English (Figure 1 A–C). The semantics of the reconstructed map are also consistent with the original map, as intuitively seen from comparison of the two color projections (Figure 8 D, E). This intuition is confirmed by numerical measures of the above defined overall correlation (**) between the original and reconstructed maps (Figure 9). In particular, altering the dimensionality of the embedding space *d*, the average number of “onyms” per color node (i.e., the average node degree), the threshold angle between “onyms”, as well as the number of color nodes, did not affect the quality of the reconstruction in a wide range of parameters. In other words, the results of this approach are robust with respect to alteration of the corpus parameters: the dimension of the embedding (Figure 9A), the number of “onyms” per “word” (Figure 9B), the number of “words” (Figure 9C), and the maximal/minimal distance or angle between “synonyms”/“antonyms” (Figure 9D).

## Discussion

In his 1946 “Man's Search for Meaning”, neurologist and psychiatrist Viktor Frankl maintained that life has meaning under any imaginable circumstance, that the search for this meaning is the core human drive, and that personal freedom consists of the individual choice of such meaning [23]. Although internal meaning may be viewed as the most (or arguably, the only) important matter of human existence, its scientific characterization has so far resisted the otherwise seemingly unstoppable strides of technological progress. This topic has been at times dismissed as metaphysical due to the perceived impossibility to reconcile the individual, first-person perspective of the very meaning of any concept, and the scientific requirements for objective validation, unambiguous communication, systematic reproducibility, and empirical falsifiability. Recently, however, the need, potential, and importance of extending traditional research paradigms to include subjective experience have been recognized with increasing urgency [24], [25]. One of the missing foundations is a precise measure of the content of mental states. The present study is a step toward bridging this gap.

### Major Conclusions

This study demonstrates the possibility to derive a precise metric system for semantics of human experiences objectively from data collected without using human subjects. More generally, the new technical approach we presented may have practical implications for multiple fields. Previous studies that resulted in semantic maps either relied on subjective human judgments (e.g. ANEW [18], semantic differential [10]) or were not explicitly related to human experiences (e.g. LSA [4], Latent Dirichlet Allocation: LDA [26]). In contrast, we constructed a prototype general metric system for semantics from all-purpose dictionaries, and validated its applicability to human experiences by available psychometric data. The significant correlation between the affective space of ANEW and our semantic cognitive map establishes a strong, novel, and unexpected connection between results in experimental psychology and computational linguistics.

Self-organizing semantic maps have been described before [27], and numerous methods exist to construct spatial representations of lexical knowledge (e.g., [28]). However, to our knowledge, this is the first objective approach to construct, based on available data, a simultaneous quantitative representation of synonymy and antonymy in a continuous metric space, whose dimensions have clearly identified general meanings. The low dimensionality of this semantic map indicates that, although thousands of distinct categories of meanings are conceivable, only very few apply to all contexts without a substantial domain-specific alteration of their semantic content. This limited number of general meanings is consistent with recent independent linguistic dimensional analyses [29] and contrasts with the extensive lists of semantic categories represented in Roget's thesaurus and related or similar endeavors [30]. At the same time, the remarkable consistency of the significant principal components of our map across dictionaries and languages, as well as with previous psychometric data obtained with very different methods (such as factor analysis and word ranking), suggests that they may be rooted in the fundamental laws of the human mind.

The three dominant semantic categories revealed in our study (“good-bad”, “calm-excited”, “open-closed”) are consistent with earlier psychometric, cognitive, and linguistic theories and findings, including Osgood's semantic differential [10] and Leary's interpersonal Circumplex [31] (cf. [32]). In particular, semantic differential rating was devised as a scale to measure the affective meaning of objects, events, and concepts. Subjects evaluate the semantic content of a term as a relative position between two bipolar words, such as warm-cold, bright-dark, beautiful-ugly, sweet-bitter, fair-unfair, brave-cowardly, meaningful-meaningless. Through factor analysis of large collections of semantic differential scales, Osgood characterized three recurring attitudes: evaluation, potency, and activity. These dimensions, mostly corresponding to the adjective pairs “good-bad”, “strong-weak”, and “active-passive”, respectively, were found to be cross-cultural universals [33]. There is a clear resemblance between these connotations and the principal semantic components of language that emerged in our approach. Similarly, the interpersonal Circumplex is a two-dimensional representation of personality based on agency, or power (status, dominance, and control), and communion, or love (solidarity, friendliness, and warmth: [34]).

The possibility to objectively define a quantitative scale for the major categories of general semantic content, capturing both synonym and antonym relations, has practical applications to linguistic data mining [26] and sentiment analysis [35]. The main scientific value of the constructed map, however, is to lay the foundation of a precise metric system for meaning that goes far beyond the current practice of qualitative assessment [36], with important implications for artificial intelligence and cognitive neuropsychology [2]. In fact, a rigorous science of mind may require a precisely defined, universal metric system for mental state semantics [2], [8]. Similarly, in cognitive architectures representations need to be sorted by their semantics [37].

### Related Works and Novelty of the Contribution of This Work

Low-dimensional vector-space representations of word meaning were constructed previously at least in two fields, namely computational linguistics and experimental psychology. In the former case (e.g. LSA [4], probabilistic latent semantic analysis, or pLSA [38], LDA [26], [38], Isomap [39]) the purpose is often to improve information retrieval systems by indicating which documents are similar and which are not. Efforts in experimental psychology (Semantic Differential [10], ANEW [18], Circumplex [32]) aim to describe aspects of human semantic memory and affective states. The present work connected results of these two fields by establishing a correspondence between the objectively constructed semantic cognitive map and ANEW [14]. Previous semantic maps created with different techniques did not demonstrate similar features. The observation that positive words are used more frequently in English than negative words provides additional evidence for the usefulness of the map as a metric system for human experiences.

The semantic similarity of our map with ANEW in the first two dimensions was quantitatively confirmed by canonical correlation analysis, based on the map locations of words that are common for the two maps. However, the two maps are not equivalent to each other. The map constructed in the present study contains more dimensions and more words, including words that do not belong to affective stimuli. Most importantly, this map differs qualitatively from previous data as it was not constructed based on given semantic dimensions. Instead, semantics of our map dimensions are emergent and defined by the locations of all words together.

The constructed semantic cognitive map provides one geometrical representation for two relations: synonymy and antonymy. Most existing automated methods infer synonymy from word co-occurrence [19] and do not explicitly account for antonymy. Thus, the ability to represent antonymy, which may capture a vital aspect of meaning [40], constitutes an essential feature of our approach. Previous semantic cognitive mapping studies involving dissimilarity metric [4], [6] had problems to find a geometric representation of antonymy (e.g., [41]). This limitation of known approaches could be due to the non-trivial relation between antonymy and the traditionally used dissimilarity metric. For example, *king* and *queen* could be synonyms, as in head of the royal family, or antonyms, as in gender (see also footnote 1 above). Our choice of energy function (*) departs from the current paradigm. The principal components of the resulting map uniquely capture the general aspects of antonymy, i.e. those that apply to most contexts. Accordingly, the notions of synonymy and antonymy used in our analysis differ from the concepts of similarity and dissimilarity as defined by co-occurrence, as illustrated by the king/queen or hot/cool examples mentioned above. Many definitions of antonymy were proposed over the years [42]–[46], and none of them is reducible to a notion of (dis)similarity.

Unlike with LSA and related techniques, were the low-dimensionality of the map results from manual truncation of higher dimensions [5], in our case this property emerged naturally. This may have broad implications. In the foundational hypothesis of a set of categories as generators of language, the number of necessary categories was believed to be large [11]. The idea that such large variety of antonymy senses used in natural language is reducible to relatively few basic notions was actually discussed in the previous century [47], but is no longer considered in modern linguistics. It is therefore surprising that this reduction can be achieved with only three or four basic dimensions.

Unlike most previous studies, our model was not tailored for a special practical purpose, but was constructed starting from basic principles. Our energy function was selected as the most parsimonious analytical expression corresponding to the concept of synonym and antonym vector alignment. The first term is the simplest analytical expression that attempts to align synonym vectors in parallel and antonym vectors in opposite directions. The last term is the lowest symmetric power term that is necessary to keep the distribution compact. This conceptual framework significantly differs from the frameworks mentioned above, including LSA [5], LDA [26], Multidimensional Scaling (MDS) [48], etc. Semantics of the principal dimensions of our map are reproduced across databases and languages. This is not a characteristic of any previously constructed vector semantic map in computational linguistics. Even though dimensions of the earlier constructed maps have identifiable semantics, those semantics are domain-specific, and there is no visible semantic similarity between our map and various vector representations of semantics of words constructed using LSA, LDA and other approaches.

### Limits and Applications of the Semantic Map

Although the constructed semantic map reveals definitive semantics in each of its significant principal components, the vector associated with every word in the map should be interpreted as a “noisy” measure rather than an exact set of numerical values. This cautious interpretation is motivated by two considerations. First, the positions of individual words on the map depend on the selection of available synonym-antonym links, which only constitute a small subset of all possible synonym-antonym links. Adding or deleting a link changes map coordinates of the corresponding words. Stated differently, any dictionary of synonyms and antonyms only provides sparse sampling of the onym graph.

The quantitative extent of this sparse sampling can be estimated by comparing two independent thesauri, such as MS English and WN English. Limiting the respective dictionaries of synonyms to the pool of their 5,926 words in common leaves 30,922 links for MS and 12,188 for WN, with 6,576 overlaps. Assuming that synonyms in each of the two dictionaries are sampled randomly and independently from the “comprehensive” set of all true synonyms, the cardinality of the true synonym set can be computed as (30,922·12,188/6,576) = 57,311. Thus, the MS and WN English dictionaries only represent at most ∼54% and 21%, respectively, of all synonyms. However, the assumption of independent random sampling is unlikely to be realistic, because more usual synonyms may have a greater chance to be listed in both dictionaries, thus increasing the number of overlaps. Therefore, these values should be considered coarse overestimates, and the real representation is likely to be even sparser.

The second major source of noise in the constructed map is that each word is associated with a number of potentially very different meanings, or “senses”. For example, the word *mean* can assume the distinct meanings of “average”, “nasty”, and “indicate”. Therefore, the word vector may be forced to find a compromise orientation that does not match precisely any of the word meanings. From this perspective, the constructed map crudely approximates meanings with words. Semantics of individual words may not match precisely semantics of their map locations, and therefore should not be taken as literal definitions of the latter. Although the map was constructed based on relations among individual words, precise numerical definitions of its semantics only apply to large subsets of words, as in the analyses involving word frequency data (Figure 8).

More generally, individual map locations can be viewed as representing unambiguous, topographically organized semantics defined by the entire distribution of all words on the map rather than by one word. In particular, the map location of a specific meaning could be computed precisely as the center of mass of the group of all its representing words. Two meanings with close/opposite centers of mass would be more likely to be synonym/antonym than two individual words separated by the same distance on the map. The accuracy of the map location of a meaning would increase with the number of its representing words. Ideally, in order to precisely allocate meaning on the map, the center of mass of all dictionary words should be computed with appropriate weights measuring their semantic agreement with the given meaning.

As a result of these two limitations, namely sparse sampling and approximation of meanings with words, individual word coordinates are subject to considerable noise, the relative amplitude of which can be roughly estimated as 10–20%. Nevertheless, the map is robust with respect to the assignments of synonyms and antonyms, and their connotation, from sets of related words. In particular, within all onyms of onyms, constituting a pool of terms likely related to a given word, dot product is a powerful predictor of semantic content (Figure 8 and Table 2). Moreover, when global map characteristics are derived from all word coordinates, as in cross-corpus map correlations (Table 3) the noise effectively averages out. This means that the map can be used as a precise semantic scale, even if individual words cannot.

In addition, our map does not capture the whole semantics of a word, but only the aspect that distinguishes between synonyms and antonyms in a context-independent query. The domain-specific part of meaning, including the aspect that determines the likelihood for a word to appear in a particular topic or document, is missed equally for all words. Words that fall near the origin (like “emigrant”) do not have a significant measure of the “semantic flavor” that this map represents. This is also why finding unrelated words next to each other on the map does not indicate an inconsistency.

### Relating the Constructed Word Map to Semantic Space

**Figure 10:**
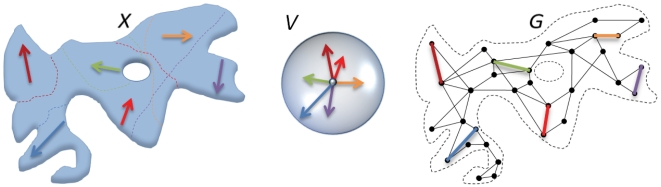
Semantic space concept. *X*: space of concepts (meanings) internally delineated by distinct domains of applicability; *V*: space of relations among concepts; *G*: graph of relations among selected concepts in *X*. Links connecting concepts in *X* and in *G* are translated to common origin in *V* and rotated to minimize the energy function (*), while preserving their consistent angular relations that correspond to the notions of synonymy and antonymy.

Semantic space, or the set *X* of all meanings, by assumption can be mapped into a high-dimensional Euclidean space (Figure 10, left). Selected relations among meanings represented by words are shown as vectors connecting points of *X* (colored arrows). These relations have each their own domain of applicability in *X*. Dashed lines of corresponding colors show the domain boundaries. For example, the word *hot* can be viewed as a label for the relation among two meanings represented by points in *X*, one of which can be considered *hot* as compared to the other: the red color is hot compared to the blue color, the weather in Mexico is hot compared to Canada, the housing market in Manhattan is hot compared to that in Detroit. The relation *hot*, however, has a limited domain of applicability. For instance, this concept does not make sense in general when referred to pairs of elementary geometrical shapes. As a particular example, a triangle can be said to be *sharp*, but not *hot*, compared to a circle.

Domains of applicability of two relations labeled by words may be overlapping or disjoint. For example, domains of applicability of *hot* and *sharp* overlap, e.g. in the food domain, while the domains of applicability of *differentiable*, a mathematical term, and *charismatic* appear to be disjoint. Two relations labeled by words within an overlap of their domains are synonyms, if their vectors point in the same or similar directions (e.g., *hot* and *sharp* in the food domain). They are antonyms, if their vectors point in the opposite or nearly opposite directions (e.g., *hot* and *cold*). These notions of synonymy and antonymy have a clear geometrical interpretation in *X* locally. However, they may or may not be globally consistent. For instance, *good* and *bad* are in general globally consistent antonyms, i.e. they point in nearly opposite directions in all of their overlapping domains of applicability. In contrast, *hot* and *cool* are often antonyms but occasionally point in similar directions, i.e. are synonyms, as in the example of “a *hot* videogame” and “a *cool* videogame” (cf. footnote 1).

The vectors representing relations labeled by words, when translated to a common origin, span a vector space *V*. Here they can be further rotated to reduce the dimension of *V*, respecting the following rule: global synonyms should remain nearly parallel and global antonyms nearly anti-parallel. However, the converse may not be true. For example, if red and brown arrows (Figure 10) have overlapping domains in *X and* represent synonyms, then they should be nearly parallel in *V*. Blue and purple arrows have disjoint domains and therefore cannot be called global synonyms or antonyms, despite the fact that they are nearly parallel in *V*. Thus, their mutual orientation in the embedding of *X* could be any. Red and purple arrows have overlapping domains and are nearly anti-parallel in *X* (antonyms), therefore, they have to keep this property in *V*. However, brown and purple arrows cannot be antonyms, because their domains are disjoint. Red and green arrows have overlapping domains in *X* and are orthogonal in their common domain in *X*: they are neither synonyms nor antonyms. While in principle according to the above rule they can be oriented at any angle in *V*, our numerical experiments show that they are more likely to be nearly orthogonal to each other in *V*, if other angular relations within the overlap of their domains are satisfied.

The above rule to translate and rotate vectors from *X* to *V* is captured by the energy function described in Materials and Methods (*). As a consequence of the optimization process, the dimension of *V* can be smaller than the dimension of the Euclidean space into which *X* is mapped. However, because metrics in *V* respect consistent synonym and antonym relations among all vectors defined at any given location in *X*, the dimension of *V* is unlikely to be smaller than the dimension of *X* itself. Therefore, the dimension of *V*, which in our analysis is ∼4 provides an approximate upper bound on the dimension of *X* and a lower bound on the dimension of the Euclidean space into which *X* is mapped.

According to this interpretation, the results of our work can be restated as the following. There are only a small number (∼4) of independent (“orthogonal”) semantic relations that generally apply in a consistent manner to almost all possible domains of applicability. In order of importance, or of the amount of meaning they express, as measured by the captured variance, they can be identified as good/bad (valence), calm/excited (arousal), open/closed (freedom), and copious/essential. The first three of these dimensions are consistent across corpora and languages.

An alternative, simplistic view of the semantic space *X* is a connected graph *G* (Figure 10, right), where nodes are words now interpreted as corresponding to broad categories in the set *X*. Edges of *G* represent relations among words, namely synonymy (black) and antonymy (colored). Because each meaning of a word, and in most cases each word, typically has at most one antonym in the dictionary, words again can be associated with directions of their antonym links and therefore can be embedded as vectors in *V*, as described above. The above analysis suggests that equivalent semantic properties of *V* will result from interpretation of either individual words or pairs of antonyms as vectors in *V*.
