# Correction: Principal Semantic Components of Language and the Measurement of Meaning

**DOI:** 10.1371/annotation/76179ada-64b5-4931-8f2d-3528f17d8359

**Published:** 2010-07-14

**Authors:** Alexei V. Samsonovich, Giorgio A. Ascoli

The first author's name is spelled incorrectly. The correct name is Alexei V. Samsonovich. The citation should read: Samsonovich AV, Ascoli GA (2010) Principal Semantic Components of Language and the Measurement of Meaning. PLoS ONE 5(6): e10921. doi:10.1371/journal.pone.0010921 

